# The colder, the better? Sustainability in frozen storage of a monoclonal antibody drug substance

**DOI:** 10.1016/j.ijpx.2026.100572

**Published:** 2026-05-20

**Authors:** Ricarda Nagel, Karoline Bechtold-Peters, Wolfgang Frieß

**Affiliations:** a Pharmaceutical Technology and Biopharmaceutics, Department of Pharmacy, Ludwig-Maximilians-Universität München, Butenandstraße 5-13, 81377 Munich, Germany; b Technical Research and Development, Novartis Pharma AG, 4002 Basel, Switzerland

**Keywords:** Frozen Storage, Drug Substance, Glass transition Temperature, Monoclonal Antibody, Stability Sustainability

## Abstract

This study investigates the long-term stability of a monoclonal antibody (mAb) in selected formulations containing histidine buffer, sucrose, and 2-hydroxypropyl-β-cyclodextrin (HPβCD). Samples were stored for up to 24 months at −80 °C, −40 °C, and − 10 °C to assess whether more energy-efficient storage temperatures can maintain drug quality. We analyzed key physical stability attributes by monitoring high-molecular-weight species, subvisible particles, and optical density, while chemical stability was assessed by monitoring methionine oxidation. A primary focus was evaluating whether physical stability in the frozen state correlates with the formulation's glass transition temperature (Tg’) as an indicator of matrix mobility. We report that storage at −40 °C and, in many cases, even at −10 °C, offers stability comparable to −80 °C, making these temperatures viable and more sustainable long-term storage options. Formulations containing HPβCD as a novel approach for freeze-thaw and frozen storage demonstrated the best stability, likely due to its combined cryoprotective and interfacial-shielding properties, which reduce both aggregation and particle formation. Data demonstrate a drastic stability change at Tg’, with substantially increased stability below Tg’. However, above and below Tg’, the temperature difference relative to Tg’ had only minor influence compared to formulation effects. We therefore hypothesize that the sharp change in matrix mobility primarily governs frozen-state stability. The observation that several formulations remained stable at −10 °C highlights that formulation-dependent mechanisms can compensate for increased matrix mobility, offering promising potential for more flexible, sustainable frozen storage conditions.

## Highlights

• Storage at −40 °C preserves mAb stability equal to −80 °C over 24 months.
• Formulation outweighed Tg’ in governing frozen-state stability of a model mAb.
• Low excipient concentrations maintain long-term frozen mAb stability up to −10 °C.
• HPβCD effectively minimizes aggregates and SVPs across all temperatures.

## Graphical Abstract

**Figure:**
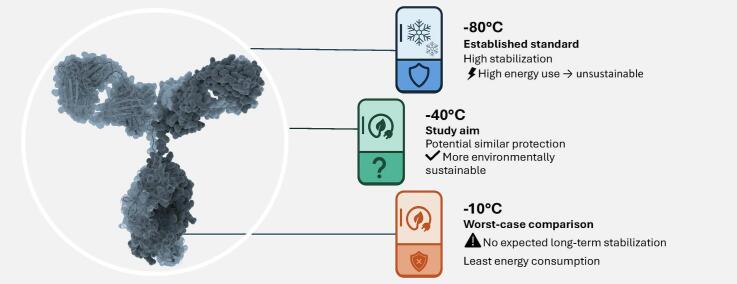

## 1 Introduction

Protein pharmaceutics have become a highly important therapeutic group, offering high target specificity and effectiveness for a wide range of diseases with minimized side effects. Their conformational, colloidal, and chemical stability is closely tied to temperature, typically making storage of both drug substance and drug product below room temperature necessary.(Authelin et al., 2020; Rosa et al., 2017; Singh et al., 2009).

Specifically, protein bulk drug substance (DS) is commonly stored frozen at ultra-low temperatures of −70 to −80 °C to prevent microbial growth, foaming, agitation during transport, and protein degradation.(Authelin et al., 2020; Bluemel et al., 2020; Rodrigues et al., 2011) The costs, logistics, and environmental impact of maintaining such conditions significantly increase the complexity of production and shipping. The main objective of this study is to evaluate and propose −40 °C as a new standard for long-term frozen storage of protein drug substances, aiming to maintain product quality while substantially improving sustainability. Implementing more sustainable storage methods offers enormous benefits. It enables the reduction of environmental impact by utilizing energy-efficient freezing and frozen storage systems, substantially minimizing carbon footprint, lowering storage costs, and simplifying logistics. To achieve this, understanding the impact and potential limitations of higher-temperature subzero storage on protein stability is crucial. To this end, we investigated the impact of the glass transition temperature (Tg') of the formulation on protein stability in the frozen state. Below Tg' the matrix mobility is restricted and stability should be enhanced. Yet whether further lowering the temperature once already below Tg' provides additional benefits, or whether other formulation factors have more influence was to be elucidated.

Despite reducing mobility, coupled reaction rates, and thus protein degradation, freezing and frozen storage pose several stresses to proteins. Upon freezing, after ice nucleation, the crystalline ice phase forms, and water molecules are progressively removed from the initial solution until a freeze-concentrated matrix (FCM) is formed, containing approximately 20–30% water and 70–80% solids.(Seifert et al., 2020; Seifert and Friess, 2020) Low temperatures and interaction with ice interfaces can lead to protein unfolding and exposure of aggregation-prone residues.(Arsiccio and Pisano, 2020; Chang et al., 1996; Gomes et al., 2021; Rosa et al., 2017; Schwegman et al., 2009; Strambini and Gabellieri, 1996) The upconcentration of the liquid phase leads to an increased ionic strength and potentially to phase separation.(Authelin et al., 2020; Bluemel et al., 2021a, Bluemel et al., 2021b; Dong et al., 2009; Hauptmann et al., 2018) Additionally, the buffer's pH may shift as temperature decreases, particularly when buffer components crystallize.(Kolhe et al., 2010; Thorat et al., 2020) Furthermore, as the temperature is lowered, the solubility of gases is enhanced, which in the case of increased dissolved oxygen may increase the risk of oxidation.(Authelin et al., 2020; Emerson et al., 1999; Shalaev and Hill, 2021; Singh et al., 2009) Further upconcentration of gases in the FCM and possible exclusion from the ice phase as microbubbles may occur. Several studies have demonstrated that freeze-concentrated oxygen and higher storage temperature accelerate protein oxidation in the frozen state. (Al-Dalali et al., 2022; Takenaka et al., 1996; Utrera et al., 2014).

As the FCM is cooled, viscosity gradually increases until the temperature falls below Tg’ at which it drastically increases by several orders of magnitude and the FCM solidifies.(Seifert and Friess, 2020) Molecular mobility becomes extremely reduced; and the likelihood of protein degradation should be substantially lower compared to the frozen state above Tg’.(Bluemel et al., 2021a; Connolly et al., 2015; Tang and Pikal, 2005) According to Gordon-Taylor, Tg’ depends on the formulation components, specifically their respective Tg’ values and their weight fraction in the mixture.(Fox and Loshaek, 1955; Her and Nail, 1994) However, as shown by Foerst et al., polyhydroxy compounds such as carbohydrates often display a sigmoidal, non-ideal Tg'–composition relationship due to nonlinear plasticization and thermal volume expansion, which together result in non-linear shifts of Tg'. The Tg’ can span over several degrees, and the FCM may not be completely immobilized at temperatures close to Tg’. Storage at temperatures near or above Tg’ has been shown to increase protein aggregation, and it is suggested that a frozen DS formulation should be stored with a safety margin of 10 °C below Tg’ to restrict molecular mobility and, thus, potential degradation over the long time of storage up to several years.(Alhalaweh et al., 2015; Singh et al., 2009).

Relatively high Tg’ values ranging from −12 to −18 °C have been reported for pure protein solutions.(Bluemel et al., 2021a; Pansare and Patel, 2016) Commonly used excipients in protein formulations, such as buffers, salts, cryoprotectants, and surfactants, can stabilize against various stresses during the freezing process but significantly lower the formulation Tg’.(Her et al., 1995; Pansare and Patel, 2016) In the absence of excipient molecules that screen protein-protein interactions, either through ionic shielding or physical separation, upconcentration will result in a higher viscosity at a given protein concentration during freezing.(Agasty et al., 2021; Colby et al., 1991; Zhang and Liu, 2017) This may be beneficial for stabilization, as vitrification is reached earlier during the freezing process, and overall resulting viscosity is higher.

Histidine, being one of the most common buffer choices, provides charge stabilization and remains amorphous during freezing, minimizing severe pH shifts compared to crystallizing buffers; however, its pKa and thus the pH still shift slightly with temperature.(Kolhe et al., 2010) In addition; molecular dynamics studies have demonstrated the potential of histidine to interact with the protein surface through direct non-covalent adsorption.(Saurabh et al., 2022) Through this mechanism; aggregation-prone hydrophobic sticky patches can be shielded. Histidine can also act as a scavenger for reactive oxygen species (ROS); potentially preventing protein oxidation during freeze-thaw.(Wade and Tucker, 1998) A buffer at pH 5.5 shows a relatively low Tg’ of −44 °C.(Bluemel et al., 2021a) Identifying an optimal low to medium excipient concentration that balances lowering of Tg' with sufficient stabilizing effect is therefore critical for frozen storage stability. Sucrose; as a common cryoprotectant; has a moderate Tg’ of −34 °C. Proposed mechanisms by which sugars stabilize proteins in the frozen state include the promotion of preferential hydration of the protein and matrix vitrification.(Arakawa et al., 2001; Arsiccio and Pisano, 2017; Bhatnagar et al., 2008; Lee and Timasheff, 1981) Additionally, they may exert a stabilizing effect due to physically separating protein molecules in an amorphous matrix. By choosing a formulation approach with minimal excipient content, a high Tg’ can be achieved while simultaneously maintaining the excipient stabilizing properties and an adequate temperature margin to enable a higher frozen storage temperature.

We investigated the stability of a therapeutic monoclonal antibody during long-term frozen storage over 24 months aiming to establish −40 °C as a more sustainable standard long-term frozen storage condition. Physical stability parameters including high molecular weight species (HMWS), optical density (OD), and subvisible particle (SVP) formation, and chemical stability in terms of methionine oxidation, were compared between the standard storage condition of −80 °C and storage at −40 °C and − 10 °C, respectively. The mAb was formulated without any stabilizer, in 0 to 20 mM histidine buffer, and unbuffered 0 to 160 mg/mL sugar formulations containing sucrose and HPβCD. Additionally, the effect of 6-month storage at −20 °C, above, yet closer to Tg’ compared −10 °C was tested. This should provide an answer to the question, whether the continuing mobility increase with rising temperature above Tg' is key for stability, or whether sufficient stabilization can be provided by the excipients, although may lead to a reduction of Tg's.We found equivalent stability at −80 °C and − 40 °C for all investigated stability parameters, regardless of formulation, making −40 °C a more sustainable alternative to conventional ultra-low-temperature storage at −70 to −80 °C.

## 2 Material and methods

### 2.1 Materials

Novartis AG (Basel, Switzerland) provided a 187.7 mg/mL stock solution of a model recombinant human IgG1 monoclonal antibody in histidine buffer at pH 5.5. L-histidine, L-histidine monochloride monohydrate, and sucrose were obtained from Merck KGaA (Darmstadt, Germany). HPβCD was supplied by Wacker Chemie AG (Burghausen, Germany). Potassium dihydrogen phosphate, dipotassium hydrogen phosphate, Dulbecco's phosphate-buffered saline 10× (pH 7.4), sodium chloride, and glacial acetic acid were purchased from VWR. FIOLAX® 2R glass vials were purchased from SCHOTT AG (Mainz, Germany) and stoppered with FluroTec® lyophilization stoppers from West Pharmaceuticals (Eschweiler, Germany).

### 2.2 Sample preparation

Formulations were prepared as listed in Table 1. The mAb stock solution was dialyzed using 30 mL Slide-A-Lyzer® cassettes from Thermo Fisher Scientific Inc. (Waltham, Massachusetts, USA) with a 30 kDa MWCO PES membrane against highly purified water (HPW) or the respective histidine buffer. An excess of 6 L target medium was used to ensure complete exchange, and the pH after exchange was measured. Subsequently, the mAb solutions were diluted to 20 mg/mL mAb with the respective histidine buffer. Samples containing sugar were prepared by dissolving the appropriate amount of sugar in mAb stock and adjusting to the final mAb concentration with buffer or HPW. The mAb concentration was determined by measuring the UV absorbance at 280 nm using a NanoDrop 2000 (Thermo Fisher Scientific, Waltham, MA, USA). Before filling, all formulations were steril filtered using 0.2 μm PES membrane syringe filters purchased from VWR. Samples were prepared in triplicate for each formulation and time point by filling 1 mL into 2R glass vials and semi-stoppering them for freezing.

**Table 1:** Overview of mAb formulations prepared at 20 mg/mL protein concentration and corresponding Tg' values of the freeze-concentrated matrix.

Buffer [mM]	Excipient	Concentration [mg/mL]		FCM Tg‘[°C]
Histidine pH 5.5	0	Sucrose	20	−25.1 ± 0.06
			40	−27.5 ± 0.13
			80	−29.3 ± 0.03
			120	−28.3 ± 0.21
			160	−28.6 ± 0.11
		HPβCD	20	−12.1 ± 0.13
			40	−13.3 ± 0.03
			80	−14.2 ± 0.07
			120	−14.4 ± 0.03
			160	−15.7 ± 1.75
	0	–	–	−16.2(Bluemel et al.; 2021a)
	5			−18.7 ± 0.17
	7.5			−19.7 ± 0.21
	10			−20.8 ± 0.34
	15			−23.5 ± 0.42
	20			−25.0 ± 0.27

The samples were frozen in a Christ Epsilon 2-6D LSCplus freeze-drier (Martin Christ Gefriertrocknungsanlagen GmbH, Osterode am Harz, Germany). Controlled nucleation via LyoCoN ice fog was applied to reduce vial to vial variability in nucleation temperature and rate. Thus differences in ice crystal size, ice–liquid interfacial area, and overall freezing behavior were to be minimized with the aim of similar ice morphology. To prevent edge effects, the sample vials were surrounded by two rows of vials containing 10% (*w*/*V*) sucrose. The vials were equilibrated at 5 °C for 60 min, cooled at 1 K/min to −5 °C, and after an isothermal hold for 60 min, controlled nucleation was induced by ice fog. Subsequently, the samples were frozen at 1 K/min to −40 °C and fully stopped.

Samples were then transferred to freezers at −80 °C (LAUDA-GFL GmbH, Burgwedel, Germany), −40 °C (Liebherr, Bulle, Switzerland), and − 10 °C (Köttermann GmbH, Uetze, Germany) for long-term storage. After storage at the respective temperature conditions for 24 h (t0), 3, 6, 12, and 24 months, triplicates of each formulation were thawed on the laboratory bench at 20 °C, and protein stability was analyzed.

### 2.3 Differential scanning calorimetry

Differential Scanning Calorimetry (DSC) was performed on a DSC 821e (Mettler Toledo, Gießen, Germany) to determine Tg’. After freezing, an additional lyophilization step was carried out to upconcentrate the 20 mg/mL formulations (see 2.2) for partial reconstitution with water. This approach was utilized to ensure a sufficient heat release signal for measuring Tg’ by increasing the mass of FCM. The final lyophilizates were reconstituted to 100 mg/mL mAb and equilibrated for 24 h. Reconstitution was reproducible, and no visible particles or incomplete dissolution were observed. Twenty microliters of the solutions were placed into an aluminum crucible (Mettler Toledo, Gießen, Germany) for measurement, with an empty crucible serving as a reference. Samples were cooled from 25 °C to −60 °C at a rate of 10 K/min. After a 1 min isothermal hold, samples were heated back to 25 °C at 3 K/min while being analyzed. Data was analyzed using the STARe Software (METTLER TOLEDO, Columbus, Ohio, USA), and Tg' was identified as the inflection point of the sigmoidal glass transition (Table 1).

### 2.4 Size-exclusion chromatography

Size- Exclusion Chromatography (SEC) was performed with an Agilent 1200 series HPLC system with a UV/Vis detector measuring at 220 nm (Agilent Technologies, Santa Clara, CA, USA) using a TSKgel G3000 SWxl column (Tosoh Bioscience GmbH, Griesheim, Germany) as stationary phase and 150 mM potassium phosphate buffer, pH 6.5, as mobile phase at 0.4 mL/min flow rate. Samples were diluted to 1 mg/mL mAb, centrifuged for 3 min at 17.000 xg with a Micro Star 17R (VWR International GmbH, Darmstadt, Germany), and 10 μL of the supernatant was injected. - Agilent OpenLAB Data Analysis Software 2.1 was used to analyze the chromatograms and quantify higher molecular weight species (HMWS) and fragments. The method variability was determined at 0.067%. Based on this variability, changes exceeding a resolution of 3xSD were considered as insignificant. Reliable quantification was assumed above 10xSD.

### 2.5 Flow imaging microscopy

Subvisible particles (SVPs) were counted with a FlowCam 8100 (Fluid Imaging Technologies, Scarborough, Maine, USA) equipped with a 10× magnification cell. VisualSpreadsheet® 4.7.6 software was used for measuring and processing. Particles were quantified using a sample volume of 160 μL, and the flow rate was set to 0.15 mL/min. The auto image frame rate was 28 frames/s with a sampling time of 60 s, and the nearest neighbor threshold for particle identification was set to 3 μm. Particle segmentation thresholds were defined as 10 for light and 13 for dark pixels. In addition, all vials were visually inspected under ambient laboratory lighting.

### 2.6 Optical density at 350 nm

Optical density at 350 nm (OD350) was determined using a Tecan Spark® multimode microplate reader (Tecan Group Ltd., Männeberg, Switzerland) with 200 μL sample in a Greiner Cellstar® 96 microwell plate (Sigma Aldrich, Gillingham, UK).

### 2.7 Analytical protein-A chromatography

Oxidation of the mAb was evaluated by protein-A chromatography, using an Agilent 1200 HPLC system to detect absorbance at 280 nm.(Loew et al., 2012) The fraction fronting the main peak reflects antibody with partial Fc methionine oxidation. A POROS™ 20 A (4.6 mmD × 50 mmL) column (Thermo Fisher Scientific Inc., Waltham, Massachusetts, USA) served as stationary phase at 23 °C*. mobile* phase A was Dulbecco's phosphate buffered saline 1× at pH 7.4, and mobile phase B was 100 mM acetic acid with 150 mM sodium chloride in HPW at pH 2.8. Elution was performed in gradient mode at 2.0 mL/min (minutes:percentage buffer B = 0:0, 40:60, 41:100, 51:100, 52:0, 62:0). Samples were centrifuged for 3 min at 17.000 xg with a Micro Star 17R, and 12.5 μL of the supernatant was injected.

## 3 Results and discussion

### 3.1 Long-term storage at −80 °C

To benchmark the best-case scenario, the physical stability of the mAb was investigated after storage at −80 °C for 3, 6, 12, and 24 months by measuring %HMWS, %LMWS, SVPs, and changes in OD350. Additionally, chemical stability concerning methionine oxidation was evaluated using Protein-A chromatography. The t0 samples were frozen, stored for 24 h, and subsequently thawed prior to analysis.

**Fig. 1:**
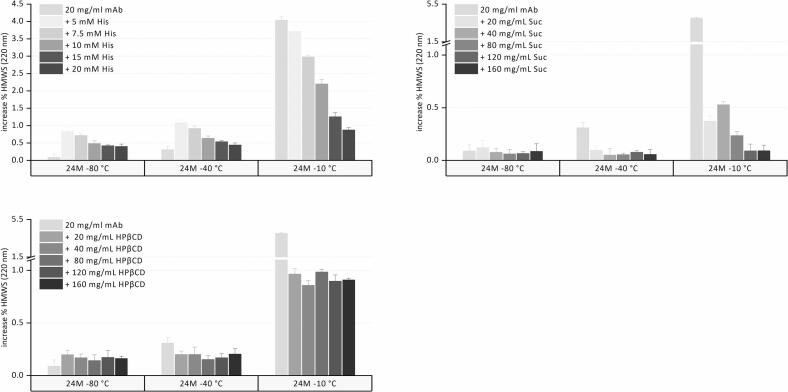
Increase in %HMWS after long-term frozen storage for 24 months at −80, −40, and − 10 °C for pure mAb, histidine, sucrose, and HPβCD formulations.

Pure mAb solution and histidine formulations exhibited a slight increase in HMWS during storage up to 6 months but remained at similar levels during further storage up to 24 months (Fig. 1). Initial HMWS levels were well above the limit of quantification (0.67%), starting at 1.24% or higher. The minor fluctuations between the different time points do not follow a distinct trend and may be attributed to slight variations in the overall handling process. HMWS formation increased with lower histidine concentration, with 5 mM histidine samples showing an increase in %HMWS of 0.97% compared to 0.40% for samples containing 20 mM histidine. Sucrose and HPβCD-containing formulations maintained their initial level of %HMWS throughout the 24-month storage period, independent of sugar concentration. The standard deviations of these samples are considerably lower compared to those of the sugar-free samples. Both excipients are sugars commonly used as cryoprotectants and they may lower the impact of slight variations during freezing and thawing on the %HMWS formation compared to formulations containing only histidine buffer.(Lee and Timasheff, 1981).

The Tg’ value of the pure mAb was −16.2 °C.(Bluemel et al., 2021a) With 5 and 20 mM histidine, Tg’ was at −18.7 °C and − 25.0 °C, respectively (Table 1). At −80 °C, no correlation was observed between Tg' and stability, indicating that once stored below Tg', the absolute difference between storage temperature and Tg' does not influence stability. Conversely, samples with higher histidine concentrations exhibited a lower Tg' yet showed reduced HMWS formation. In addition, the HMWS levels of samples containing sucrose or HPβCD remained at the initial values and were independent of the stabilizer concentration, despite different Tg’ value.

**Fig. 2:**
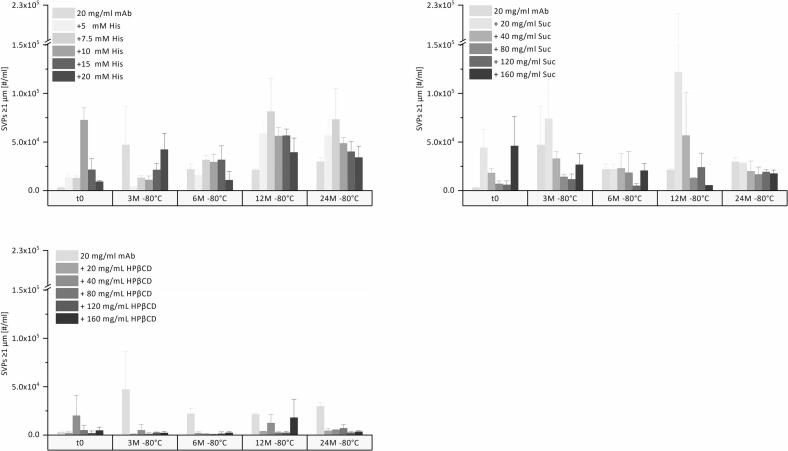
SVPs ≥1 μm/mL upon 24-month storage at −80 °C for the mAb in formulation with different concentrations of histidine, sucrose, and HPβCD.

The number of subvisible particles (SVPs) per mL (≥1 μm) in pure mAb solution and histidine buffer-containing samples is shown in Fig. 2. Pure mAb solution showed 3000 SVPs/mL (≥1 μm) at t0, which increased to 50,000 SVPs/mL (≥1 μm) after 3 months of storage but did not increase significantly thereafter up to 24 months. Samples containing histidine buffer exhibit a slight trend towards increased SVP formation during storage, independent of histidine concentration. Initial counts for 5, 7.5, 15, and 20 mM histidine formulations ranged from approximately 10,000-20,000, increasing to approximately 35,000–75,000 SVPs/mL (≥1 μm) after 24 months. For the 10 mM histidine samples, a high t0 value of 73,000 SVPs/mL (≥1 μm) was observed and did not increase over 24 months. For sucrose samples, SVPs at t0 ranged from 6000 to 46,000 SVPs/mL (≥1 μm) and did not increase over 24 months.

Consistent with these findings, no visible particles were observed upon visual inspection at any time point. Subvisible particle counts showed some variability between time points. However, no clear time dependent increase was observed upon storage, indicating limited net SVP formation under the conditions studied. Among all formulations, HPβCD samples consistently exhibited the lowest SVP levels (2000–7000 SVPs/mL (≥1 μm) at t0) and the smallest variability, and no increase was observed upon storage at −80 °C. Besides acting as a cryoprotectant, similar to sucrose, HPβCD may stabilize proteins by hosting aggregation-prone hydrophobic residues within its hydrophobic cavity and by preventing adsorption to interfaces.(Matilainen et al., 2008; Serno et al., 2010) This shielding of the mAb molecules from the ice-liquid interface formed during freezing can well explain the low SVP counts in the samples.

Regarding larger SVPs of ≤10 μm and ≤ 25 μm, consistently low SVP counts were observed across all formulations. For pure mAb solution, SVP counts increased from an initial value of 150 to 2000 SVPs/mL (≥10 μm), and from 30 to 160 SVPs/mL (≥25 μm) after storage at −80 °C for 24 months. For histidine samples, SVP numbers ranged from 0 to 300 SVPs/mL (≥10 μm), and from 0 to 50 SVPs/mL (≥25 μm) initially, increasing only slightly to 2000–6000 SVPs/mL (≥10 μm), and 700–1500 SVPs/mL (≥25 μm). Sucrose formulations exhibited initial values ranging from 200 to 2000 SVPs/mL (≥10 μm) and from 60 to 400 SVPs/mL (≥25 μm). HPβCD again exhibited the lowest initial counts, ranging from 30 to 150 SVPs/mL (≥10 μm) and from 0 to 40 SVPs/mL (≥25 μm). Neither the sucrose nor the HPβCD formulations showed an increase after 24 months of long-term storage at −80 °C.

The OD350 indicates the presence of aggregates, especially in the higher-nm size range. The values were low, ranging from 0.11 to 0.16 for all formulations, regardless of storage time and the presence of excipients.

**Fig. 3:**
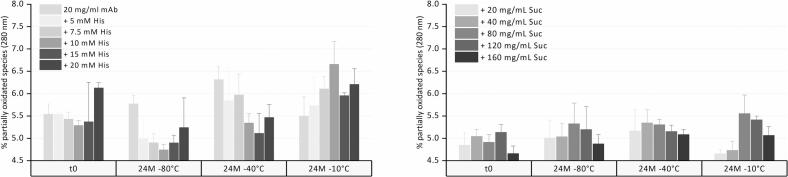
Oxidation levels upon 24-month storage at −80, −40, and − 10 °C for the mAb in formulation with different concentrations of histidine and sucrose.

Oxidation could only be evaluated in pure mAb, histidine, and sucrose samples. HPβCD interfered with the method, an effect that manifested over storage. The model mAb used did not show distinct peaks corresponding to Met252 and Met428 oxidation; instead, a shoulder in front of the main peak indicates partially oxidized species.(Loew et al., 2012) Oxidation levels ranged from 4.7 to 6.1% at t0, independent of buffer or sucrose concentration (Fig. 3). Over a 24-month period, the content of oxidized species did not change significantly.

In summary, minimal aggregate and particle formation was observed across all formulations, confirming that −80 °C provides excellent long-term mAb stability. Sucrose and HPβCD, regardless of concentration, were effective in maintaining initial HMWS levels and in producing an overall lower SVP with less variability than sugar-free samples. No correlation was found between Tg’ and storage stability; samples with higher histidine concentrations had a lower Tg’ and showed less HMWS formation.

### 3.2 Long-term storage at −40 °C

Following the study's aim, samples were also stored at −40 °C, a more sustainable temperature than −80 °C, but still below the Tg’ of all investigated formulations (Table 1). Tg’ is considered a critical temperature threshold for stabilizing the protein through solidification of the amorphous matrix.

The HMWS content increased slightly over 6 months of storage, with no further increase up to 24 months. This change in %HMWS is comparable to the one upon storage at −80 °C. However, a more noticeable increase in %HMWS occurs in samples with lower amounts of histidine buffer. For 5 mM and 7.5 mM histidine, %HMWS increased by about 0.8 and 0.7% at −80 °C, respectively, while at −40 °C the increase was approximately 1.1 and 0.9%, respectively. At higher histidine concentrations, the increase is equivalent at both storage temperatures. Pure mAb solution showed a negligible increase of only 0.1% at −80 °C compared to 0.6% at −40 °C. Formulations containing sucrose and HPβCD did not show an increase in %HMWS over 24-month storage at-40 °C, like they did upon −80 °C storage. Thus, although stored at a higher frozen temperature of −40 °C compared to −80 °C, the stabilization for any formulation remained essentially unchanged and was essentially independent of the temperature difference between storage temperature and Tg’.

**Fig. 4:**
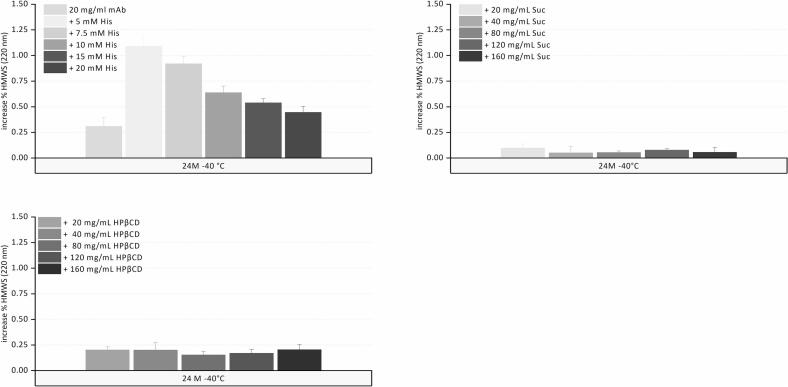
SVPs ≥1 μm/mL upon 24-month storage at −80, −40, and − 10 °C for the mAb in formulation with different concentrations of histidine, sucrose and HPβCD.

After 24 months, SVP counts (≥1 μm) were comparable for samples stored at −80 °C and − 40 °C across all formulations (Fig. 4). HPβCD formulations showed the lowest particle levels (1000 to 5000 SVPs/mL (≥1 μm)), whereas pure mAb and both histidine (15,000 to 50,000 SVPs/mL (≥1 μm)) and sucrose formulations (10,000 to 100,000 SVPs/mL (≥1 μm)) showed a broader range. The same qualitative trend and temperature independence were observed for particle ≥10 μm and ≥ 25 μm. In addition, visual inspection confirmed the absence of visible particles in samples stored at −40 °C.

The OD350 of all formulations also stayed low at values of 0.12 to 0.16 during storage at −40 °C. Additionally, the oxidation levels remained unchanged over storage at −40 °C for 24 months (Fig. 3). Therefore, storage stability is essentially equivalent at −40 °C and − 80 °C.

### 3.3 Long-term storage at −10 °C

To trigger degradation during frozen storage and to identify all critical signs of instability, formulations were stored at −10 °C. Elevated temperatures above Tg’ may also potentially occur for shorter periods during temperature excursions upon storage or shipping. The temperature of −10 °C is above the Tg’ value of all formulations (Table 1), which range from −29.3 °C in the case of the 80 mg/mL sucrose-containing formulation up to −12.1 °C for the 20 mg/mL HPβCD sample. Under these conditions, stabilization through complete solidification is no longer effective, yet molecular movement in the viscous FCM is hindered in comparison to a liquid environment.(Seifert and Friess, 2020) Signs of both physical and chemical protein degradation; as well as the impact of formulation; are expected to be amplified under these conditions.(Franks, 1998).

The formation of HMWS in pure mAb and histidine formulations was drastically increased at −10 °C (Fig. 1). In contrast to the results at −80 and − 40 °C, the increase was most pronounced for pure mAb solution with 4.0%, while increasing histidine concentration improved the stability. This finding differs from earlier reports by Bluemel et al., who observed more pronounced HMWS formation at higher histidine concentrations and a minor effect of mAb concentration.(Bluemel et al., 2021a, Bluemel et al., 2021b) A pH shift in the buffer is less likely to be the cause; given the minor change in histidine pKa at lower temperatures and the self-buffering property of the mAb at higher concentration.(Gokarn et al., 2008; Kolhe et al., 2010) Compared to Bluemel et al., we utilized a higher mAb concentration, thereby defining a different formulation regime in which reducing the histidine concentration decreased stability. Furthermore, it has been reported that a higher mAb concentration positively influences stability. As the interface between ice and the FCM becomes saturated, degradation of excess protein is limited. Thus, the degradation of frozen protein solutions relative to the total amount of protein decreases with a higher FCM mass-to-ice ratio.(Jiang and Nail, 1998; Sarciaux et al., 1999) Our finding that higher histidine concentrations improve storage stability in the frozen system aligns with observations that they inhibit monomer loss and reduce protein aggregation.(Chen et al., 2003; Kalonia et al., 2016) Molecular dynamics simulations have suggested that histidine can engage in dynamic, non-covalent interactions with surface-exposed hydrophobic regions of monoclonal antibodies, providing a qualitative mechanistic hypothesis for its potential stabilizing role.(Saurabh et al., 2022).

Ultimately, the positive effect of histidine in our study may be due to its influence on mAb self-interaction and to a higher mAb-to-histidine ratio than in Bluemel et al. Taken together, these findings demonstrate that, above Tg', histidine effects on frozen-state stability are non-monotonic and depend on the specific formulation regime rather than on concentration or ratio alone.

Formulations containing sucrose showed only minor increases in %HMWS of less than 0.5% after storage at −10 °C over 24 months, and higher sucrose concentrations up to 120 and 160 mg/mL resulted in less HMWS (Fig. 1). In HPβCD formulations, the percentage of HMWS increases to about 1%, and increasing the concentration of HPβCD does not further reduce this level.

The SVP counts in the pure mAb formulation increased from 3000 to 65,000 SVPs/mL (≥1 μm) (Fig. 4). For formulations containing 5 mM, 7.5 mM, and 20 mM histidine, the counts ranged from approximately 25,000 to 75,000 SVPs/mL (≥1 μm), slightly higher compared to storage at −80 °C or − 40 °C. In sucrose- or HPβCD-containing formulations, particle counts were approximately 20,000 to 60,000 SVPs/mL (≥1 μm) and 3000 to 5000 SVPs/mL (≥1 μm) after 24 months at −10 °C, respectively. HPβCD, already at low concentration, efficiently reduces interface related SVP formation in mAb formulations during long-term frozen storage, yet it is less efficient in overall cryoprotection compared to sucrose.(Haeuser et al., 2020) The maximum stabilization is already achieved at the lowest concentration of 20 mg/mL HPβCD.

Overall, the counts remained low during storage above Tg’. No increase in larger SVPs ≤10 μm and ≤ 25 μm was observed in any formulation, and particle counts in samples stored at −10 °C were comparable to those stored at −80 °C and − 40 °C over 24 months. Despite elevated temperature stress, no visible particles were detected by visual inspection across all formulations.

OD350 of all formulations stayed low at values of 0.12 to 0.17 during storage at −10 °C. Oxidation levels remained unchanged after storage at −10 °C for 24 months.

Overall, our results do not show a simple link between stability and the formulation Tg’, even when Tg’ is exceeded. On the one hand, the formulation with 20 mM histidine has the smallest increase in %HMWS among all histidine formulations, despite having already surpassed Tg’ by a large margin at −10 °C. On the other hand, formulations with either a lower or higher Tg’, such as 80 mg/mL sucrose or 20 mg/mL HPβCD, respectively, demonstrate even lower levels of aggregates. During frozen storage, multiple stabilization mechanisms can prevent protein degradation. This includes solidification. In addition, while buffers and ions like histidine can stabilize proteins by charge shielding, cryoprotectants like sucrose maintain protein structure through preferential exclusion and HPβCD provides additional interfacial protection.(Arakawa et al., 2001; Bhatnagar et al., 2008; Chen et al., 2003; Kalonia et al., 2016; Lee and Timasheff, 1981; Matilainen et al., 2008; Saurabh et al., 2022; Serno et al., 2010) For the pure mAb solution, the most significant increase in %HMWS was observed at −10 °C. However, we found that even low amounts of buffer or sugars were already sufficient to prevent those instabilities, regardless of their influence on Tg’.

### 3.4 Comparison of stability during frozen storage at −10 °C to −20 °C

Lowering the temperature after freezing, the viscosity of the FCM increases gradually until Tg’ is reached. The viscosity of the FCM is substantially influenced by its composition. At Tg’ viscosity increases sharply by several orders of magnitude within a narrow temperature range, drastically restricting molecular mobility. While stability may mainly be governed by the overall very low mobility below Tg’, formulation factors may be more relevant above Tg’. Some stress factors that are discussed in the context of frozen protein storage, such as cold denaturation or freezing induced pH shift, may become more relevant as temperature approaches Tg'.(Kolhe et al., 2010; Lazar et al., 2010; Privalov, 1990) However, experimental evidence for cold denaturation of mAbs is limited and strongly molecule dependent, and its contribution to instability cannot be generally assumed. (Lazar et al., 2010) It is generally advised to store with a safety margin of about 10 °C below Tg’.(Singh et al., 2009) Since substantial HMWS formation was observed at −10 °C for pure mAb and with 5 to 20 mM histidine; we additionally compared mAb stability over a 6-month period at −20 °C to separate formulation effects from an expected viscosity-driven reduction in mobility associated with storing closer to Tg'.(Seifert and Friess, 2020).

**Fig. 5:**
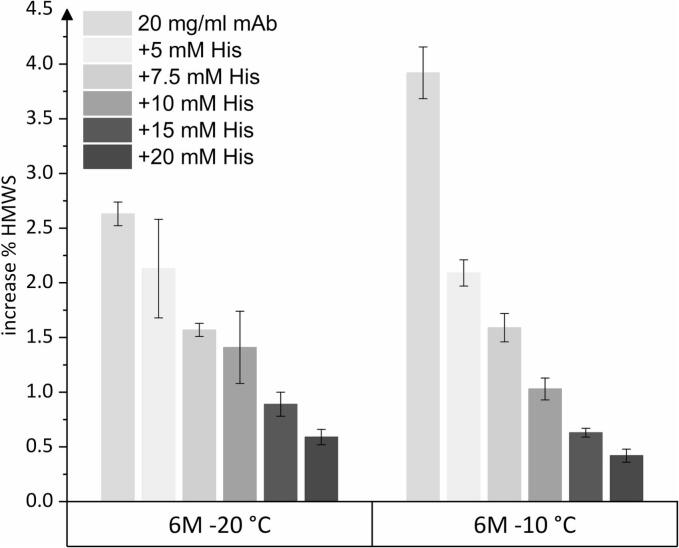
Increase in %HMWS after 6 month storage in comparison for −20 and − 10 °C for the mAb in formulation with different concentrations of histidine.

The %HMWS increased by about 2.6% in pure mAb solution stored at −20 °C below the Tg’ of −16.2 °C, compared to 3.9% when stored at −10 °C (Fig. 5). For the 10, 15, and 20 mM histidine buffer formulations, the Tg’ values were − 20.8 °C, −23.5 °C, and − 25.0 °C, respectively, meaning that these samples were at or above their Tg’ when stored at −20 °C. Despite this fact, the increase in HMWS was similar at −20 °C and − 10 °C. SVPs ≥1, ≥10, and ≥ 25 μm remained at consistent levels at both temperatures. Storage at −20 °C provided better stability than storage at −10 °C for the pure mAb, which can be attributed to solidification below Tg’ as a stabilizing mechanism in the absence of stabilizing excipients. In contrast, histidine-containing samples did not differ in stability, regardless of whether they were stored above or below Tg’. This demonstrates that, above Tg’, it was the formulation itself that had a decisive impact on the stability outcome, rather than the expected relatively small change in matrix mobility as temperature increased from −20 °C to −10 °C.

## 4 Conclusion

This study aimed to characterize more sustainable long-term frozen storage of a model mAb in different formulations at −40 °C below their Tg’ compared to the standard ultra-low temperature storage at −70 to 80 °C. Additionally, long-term storage around and well above Tg’ at −20 °C and − 10 °C was investigated to induce degradation processes and exacerbate signs of instability. These temperatures would enable an even more sustainable storage or shipment for shorter time frames. To comprehensively assess stability, we investigated both physical and chemical parameters. The model mAb was formulated at a constant concentration and pH without excipient, in low histidine/mAb ratios, and with varying concentrations of sucrose and HPβCD. Physical stability was evaluated by measuring HMWS, SVPs, turbidity, and visual inspection. Chemical stability was assessed in terms of oxidation by Protein-A chromatography.

We found that storage at −40 °C preserved stability as effectively as storage at −80 °C. At −40 °C, all tested formulations were at least 10 °C below Tg’ and under this condition, stability was not related to Tg’. Instead, samples with higher histidine concentrations had a lower Tg’, yet they were more stable, both below and above Tg’. This indicates that vitrification provides robust stabilization for this mAb, regardless of formulation.

At both −10 °C and − 20 °C, temperatures above the Tg’ for most of the histidine formulations, similar levels of physical stability were observed. Instability was pronounced at −10 °C in pure mAb and significantly improved with storage at −20 °C below Tg’. The addition of a histidine buffer, which affects mAb self-interaction through charge shielding, of sucrose, which provides stabilization by preferential exclusion, or of HPβCD, which offers interface protection, enabled effective stabilization even above Tg’. This suggests that an appropriate formulation can compensate for the lack of vitrification and achieve comparable long-term stability. This promising finding needs further investigation to determine whether formulation strategies can reliably replace storage below Tg’ in broader applications. However, even a minimal formulation with low amounts of buffer or sugars can enable long-term storage above Tg’ with no detrimental effect on stability, as the formulation can effectively compensate for stresses present in a mobile FCM. Across all storage conditions, visual inspection supported analytical results, as no visible particles were observed in any vial.

In future studies, even lower excipient concentrations and their combinations could be explored to identify an optimal minimal formulation that offers sufficient stabilization during long-term bulk frozen storage above −80 °C. A minimal formulation approach maximizes the operational space for formulation development and drug product manufacturing while ensuring effective protein stabilization at −40 °C or even up to −10 °C. Tests with additional model mAbs and other protein formats are mandatory to widen the knowledge space on long-term frozen storage at −40 °C. In conclusion, we found the same stability for a mAb in various formulations when stored at −40 °C and − 80 °C for up to 24 months which opens the avenue to a sustainable and more flexible storage and transport for bulk drug substance and potentially drug product in clinical testing.

## CRediT authorship contribution statement

**Ricarda Nagel:** Methodology, Investigation, Data curation, Conceptualization, Writing – original draft. **Karoline Bechtold-Peters:** Supervision, Resources. **Wolfgang Frieß:** Supervision, Conceptualization. **Wolfgang Friess:** Writing – review & editing.

## Declaration of competing interest

The authors declare that they have no known competing financial interests or personal relationships that could have appeared to influence the work reported in this paper.

## Data Availability

Data will be made available on request.
